# Supplementary material for: The interactome of intact mitochondria by cross-linking mass spectrometry provides evidence for coexisting respiratory supercomplexes
Source: Mol Cell Proteomics. 2017 Dec 8;17(2):216–32. doi: 10.1074/mcp.RA117.000470 (PMC5795388; doi:10.1074/mcp.RA117.000470)
Supplement: Supplemental Data [file supp_RA117.000470_133922_0_supp_23978_fzffwf.zip › spectra_annotation/mito_spectra_annotation/454-1-12-1-16-1.pdf]

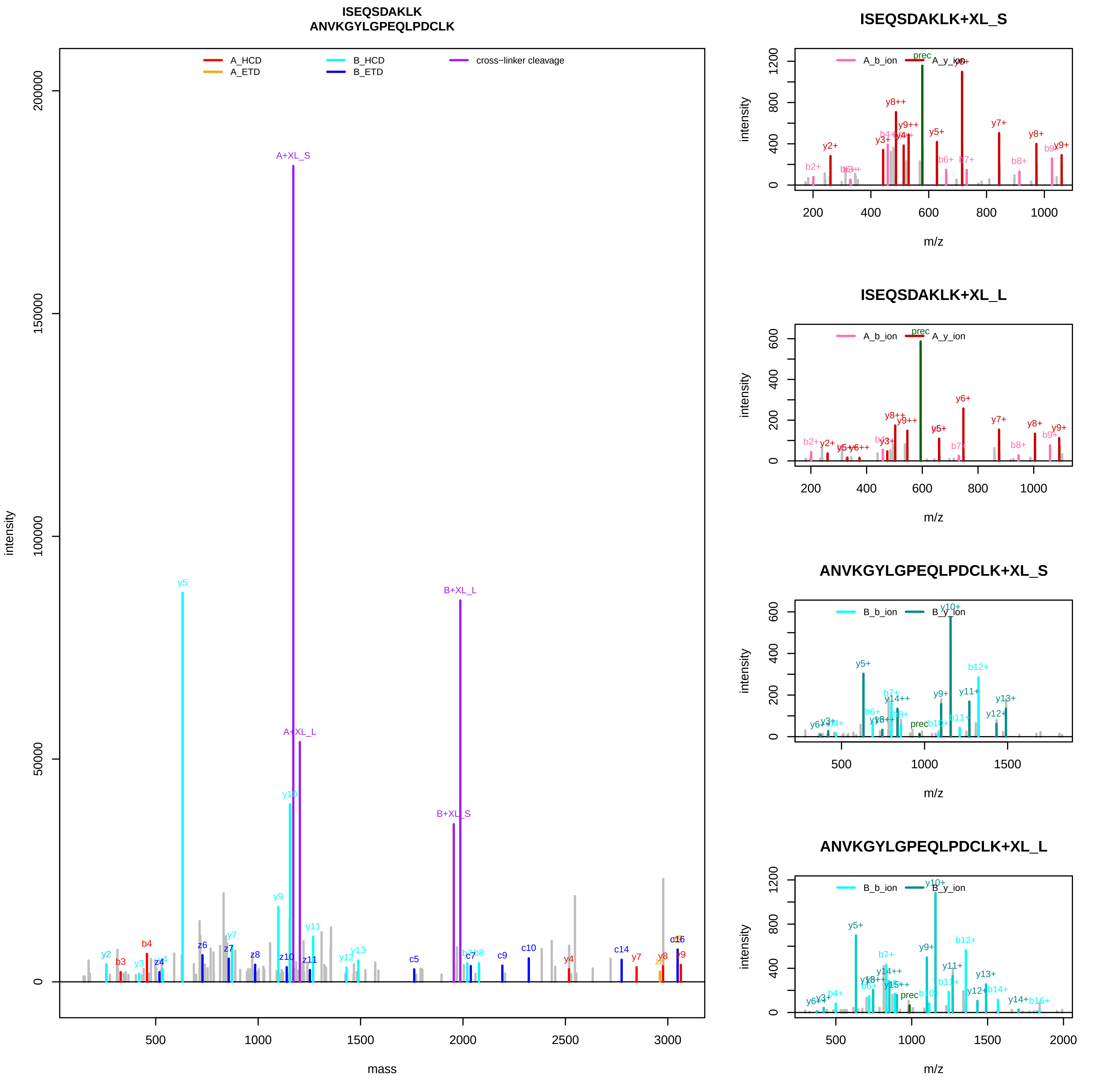
ISEQSDAKLK
ANVKGYLGPEQLPDCLK
A_HCD
A_ETD
B_HCD
B_ETD
cross−linker cleavage
intensity
mass
A+XL_S
A+XL_L
B+XL_L
B+XL_S
y5
y10
y9
y11
y13
y12
y7
z6
z7
z8
z10
z11
z4
b4
b3
y2
y3
c5
c7
c9
c10
c14
c16
b7
b8
y4
y7
y8
y9
z8
ISEQSDAKLK+XL_S
A_b_ion
A_y_ion
prec
m/z
ISEQSDAKLK+XL_L
ANVKGYLGPEQLPDCLK+XL_S
B_b_ion
B_y_ion
ANVKGYLGPEQLPDCLK+XL_L
